# Supplementary material for: The Evaluation of Bacterial Abundance and Functional Potentials in the Three Major Watersheds, Located in the Hot Spring Zone of the Tatun Volcano Group Basin, Taiwan
Source: Microorganisms. 2022 Feb 23;10(3):500. doi: 10.3390/microorganisms10030500 (PMC8949176; doi:10.3390/microorganisms10030500)
Supplement: Supplementary file 1 [file microorganisms-10-00500-s001.zip › microorganisms-1552057-supplementary.pdf]

Supplementary information

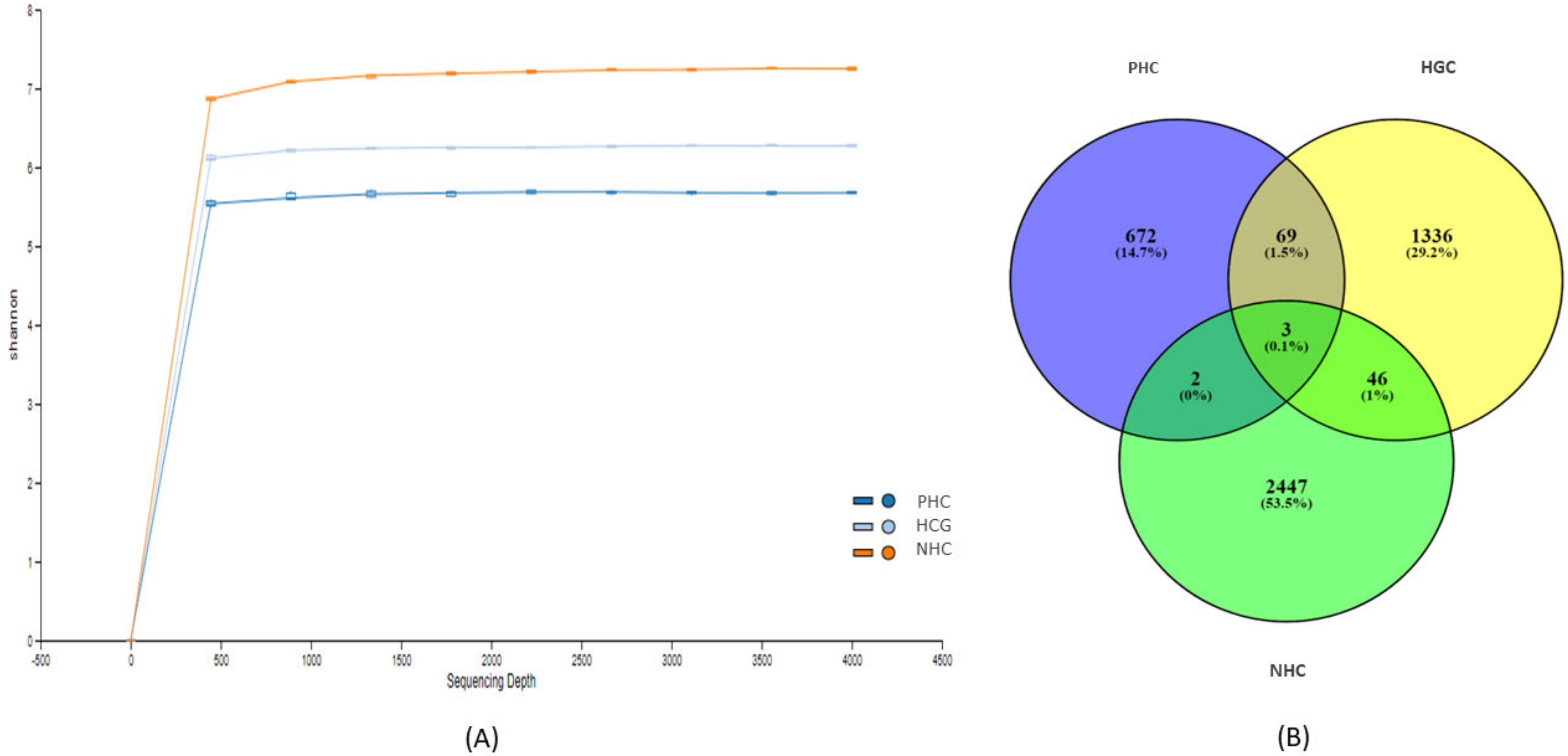

Figure S1. (A) Rarefaction plot among the samples of three watersheds (B) Venn diagram shows the proportions of unique and common/shared ASVs among the three watersheds

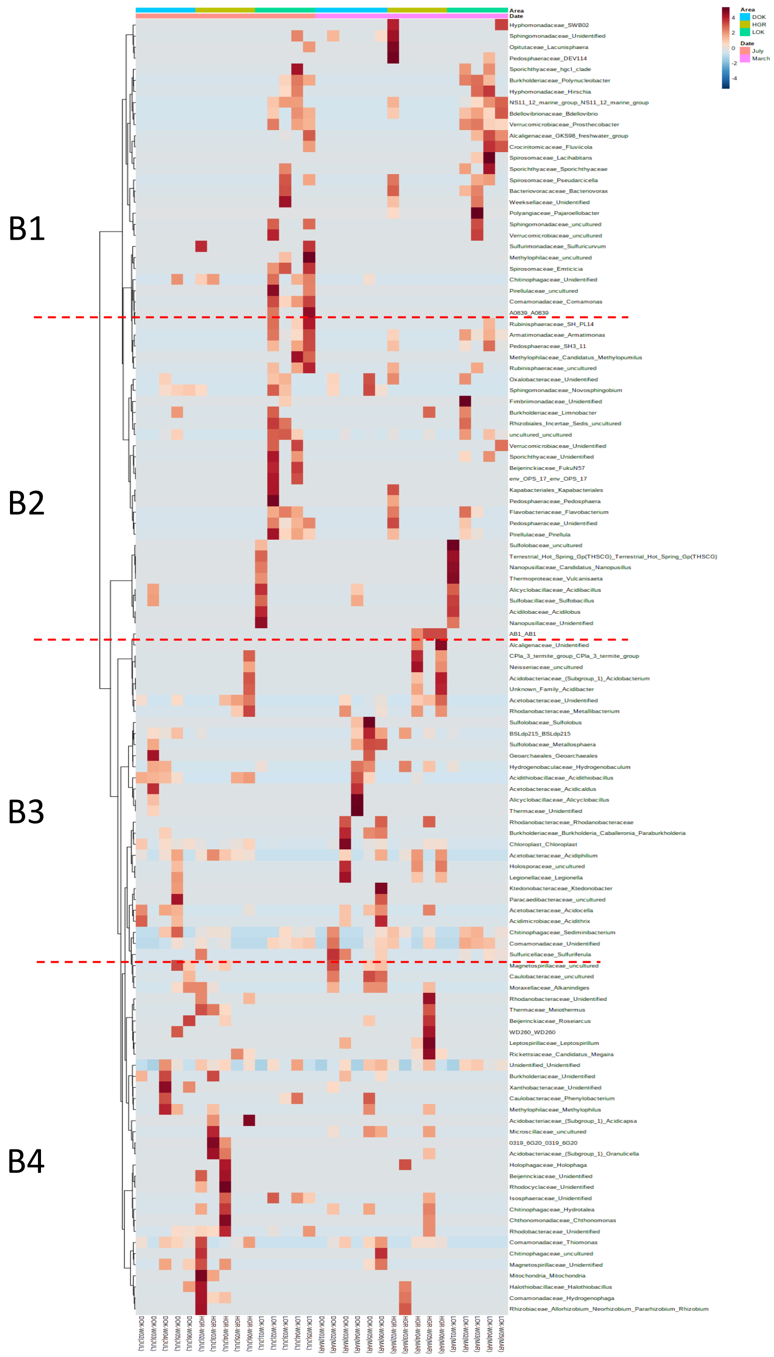

B1
B2
B3
B4
Area
Date
DOK
HGR
LOK
July
March
4
2
0
-2
-4
Hyphomonadaceae_SWB02
Sphingomonadaceae_Unidentified
Opitutaceae_Lacunisphaera
Pedosphaeraceae_DEV114
Sporichthyaceae_hgcI_clade
Burkholderiaceae_Polynucleobacter
Hyphomonadaceae_Hirschia
NS11_12_marine_group_NS11_12_marine_group
Bdellovibrionaceae_Bdellovibrio
Verrucomicrobiaceae_Prosthecobacter
Alcaligenaceae_GKS98_freshwater_group
Crocinitomicaceae_Fluviicola
Spirosomaceae_Lacihabitans
Sporichthyaceae_Sporichthyaceae
Spirosomaceae_Pseudarcicella
Bacteriovoracaceae_Bacteriovorax
Weeksellaceae_Unidentified
Polyangiaceae_Pajaroellobacter
Sphingomonadaceae_uncultured
Verrucomicrobiaceae_uncultured
Sulfurimonadaceae_Sulfuricurvum
Methylophilaceae_uncultured
Spirosomaceae_Emticicia
Chitinophagaceae_Unidentified
Pirellulaceae_uncultured
Comamonadaceae_Comamonas
A0839_A0839
Rubinisphaeraceae_SH_PL14
Armatimonadaceae_Armatimonas
Pedosphaeraceae_SH3_11
Methylophilaceae_Candidatus_Methylopumilus
Rubinisphaeraceae_uncultured
Oxalobacteraceae_Unidentified
Sphingomonadaceae_Novosphingobium
Fimbriimonadaceae_Unidentified
Burkholderiaceae_Limnobacter
Rhizobiales_Incertae_Sedis_uncultured
uncultured_uncultured
Verrucomicrobiaceae_Unidentified
Sporichthyaceae_Unidentified
Beijerinckiaceae_FukuN57
env_OPS_17_env_OPS_17
Kapabacteriales_Kapabacteriales
Pedosphaeraceae_Pedosphaera
Flavobacteriaceae_Flavobacterium
Pedosphaeraceae_Unidentified
Pirellulaceae_Pirellula
Sulfolobaceae_uncultured
Terrestrial_Hot_Spring_Gp(THSCG)_Terrestrial_Hot_Spring_Gp(THSCG)
Nanopusillaceae_Candidatus_Nanopusillus
Thermoproteaceae_Vulcanisaeta
Alicyclobacillaceae_Acidibacillus
Sulfobacillaceae_Sulfobacillus
Acidilobaceae_Acidilobus
Nanopusillaceae_Unidentified
AB1_AB1
Alcaligenaceae_Unidentified
CPla_3_termite_group_CPla_3_termite_group
Neisseriaceae_uncultured
Acidobacteriaceae_(Subgroup_1)_Acidobacterium
Unknown_Family_Acidibacter
Acetobacteraceae_Unidentified
Rhodanobacteraceae_Metallibacterium
Sulfolobaceae_Sulfolobus
BSLdp215_BSLdp215
Sulfolobaceae_Metallosphaera
Geoarchaeales_Geoarchaeales
Hydrogenobaculaceae_Hydrogenobaculum
Acidithiobacillaceae_Acidithiobacillus
Acetobacteraceae_Acidicaldus
Alicyclobacillaceae_Alicyclobacillus
Thermaceae_Unidentified
Rhodanobacteraceae_Rhodanobacteraceae
Burkholderiaceae_Burkholderia_Caballeronia_Paraburkholderia
Chloroplast_Chloroplast
Acetobacteraceae_Acidiphilium
Holosporaceae_uncultured
Legionellaceae_Legionella
Ktedonobacteraceae_Ktedonobacter
Paracaedibacteraceae_uncultured
Acetobacteraceae_Acidocella
Acidimicrobiaceae_Acidithrix
Chitinophagaceae_Sediminibacterium
Comamonadaceae_Unidentified
Sulfuricellaceae_Sulfuriferula
Magnetospirillaceae_uncultured
Caulobacteraceae_uncultured
Moraxellaceae_Alkanindiges
Rhodanobacteraceae_Unidentified
Thermaceae_Meiothermus
Beijerinckiaceae_Roseiarcus
WD260_WD260
Leptospirillaceae_Leptospirillum
Rickettsiaceae_Candidatus_Megaira
Unidentified_Unidentified
Burkholderiaceae_Unidentified
Xanthobacteraceae_Unidentified
Caulobacteraceae_Phenylobacterium
Methylophilaceae_Methylophilus
Acidobacteriaceae_(Subgroup_1)_Acidicapsa
Microscillaceae_uncultured
0319_6G20_0319_6G20
Acidobacteriaceae_(Subgroup_1)_Granulicella
Holophagaceae_Holophaga
Beijerinckiaceae_Unidentified
Rhodocyclaceae_Unidentified
Isosphaeraceae_Unidentified
Chitinophagaceae_Hydrotalea
Chthonomonadaceae_Chthonomonas
Rhodobacteraceae_Unidentified
Comamonadaceae_Thiomonas
Chitinophagaceae_uncultured
Magnetospirillaceae_Unidentified
Mitochondria_Mitochondria
Halothiobacillaceae_Halothiobacillus
Comamonadaceae_Hydrogenophaga
Rhizobiaceae_Allorhizobium_Neorhizobium_Pararhizobium_Rhizobium
LOK-W02(MAR)
LOK-W03(MAR)
LOK-W04(MAR)
LOK-W05(MAR)

2(A)

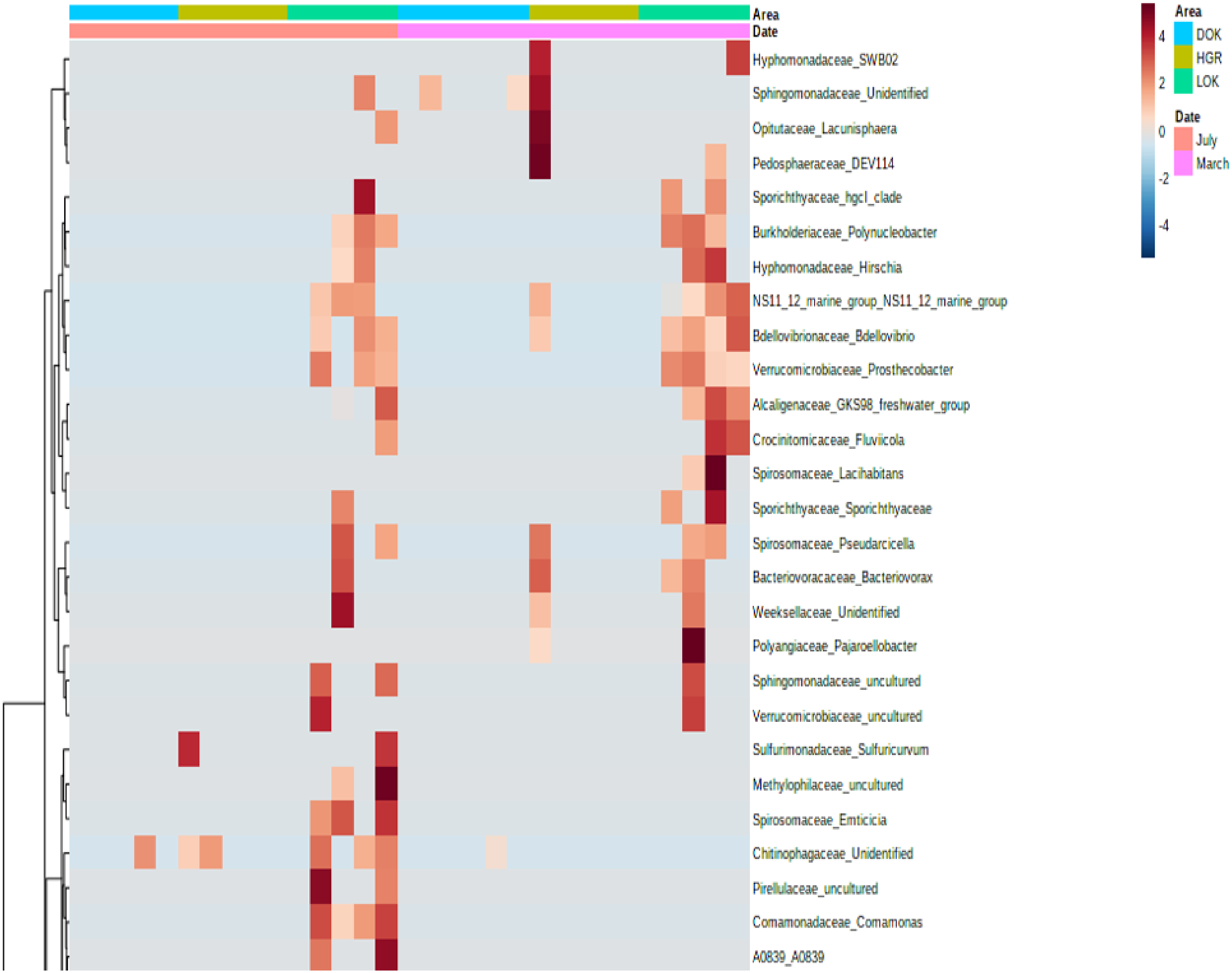

Area
Date
Hyphomonadaceae_SWB02
Sphingomonadaceae_Unidentified
Opitutaceae_Lacunisphaera
Pedosphaeraceae_DEV114
Sporichthyaceae_hgcI_clade
Burkholderiaceae_Polynucleobacter
Hyphomonadaceae_Hirschia
NS11_12_marine_group_NS11_12_marine_group
Bdellovibrionaceae_Bdellovibrio
Verrucomicrobiaceae_Prosthecobacter
Alcaligenaceae_GKS98_freshwater_group
Crocinitomicaceae_Fluviicola
Spirosomaceae_Lacihabitans
Sporichthyaceae_Sporichthyaceae
Spirosomaceae_Pseudarcicella
Bacteriovoracaceae_Bacteriovorax
Weeksellaceae_Unidentified
Polyangiaceae_Pajaroellobacter
Sphingomonadaceae_uncultured
Verrucomicrobiaceae_uncultured
Sulfurimonadaceae_Sulfuricurvum
Methylophilaceae_uncultured
Spirosomaceae_Emticicia
Chitinophagaceae_Unidentified
Pirellulaceae_uncultured
Comamonadaceae_Comamonas
A0839_A0839
4
2
0
-2
-4
Area
DOK
HGR
LOK
Date
July
March

2(B1)

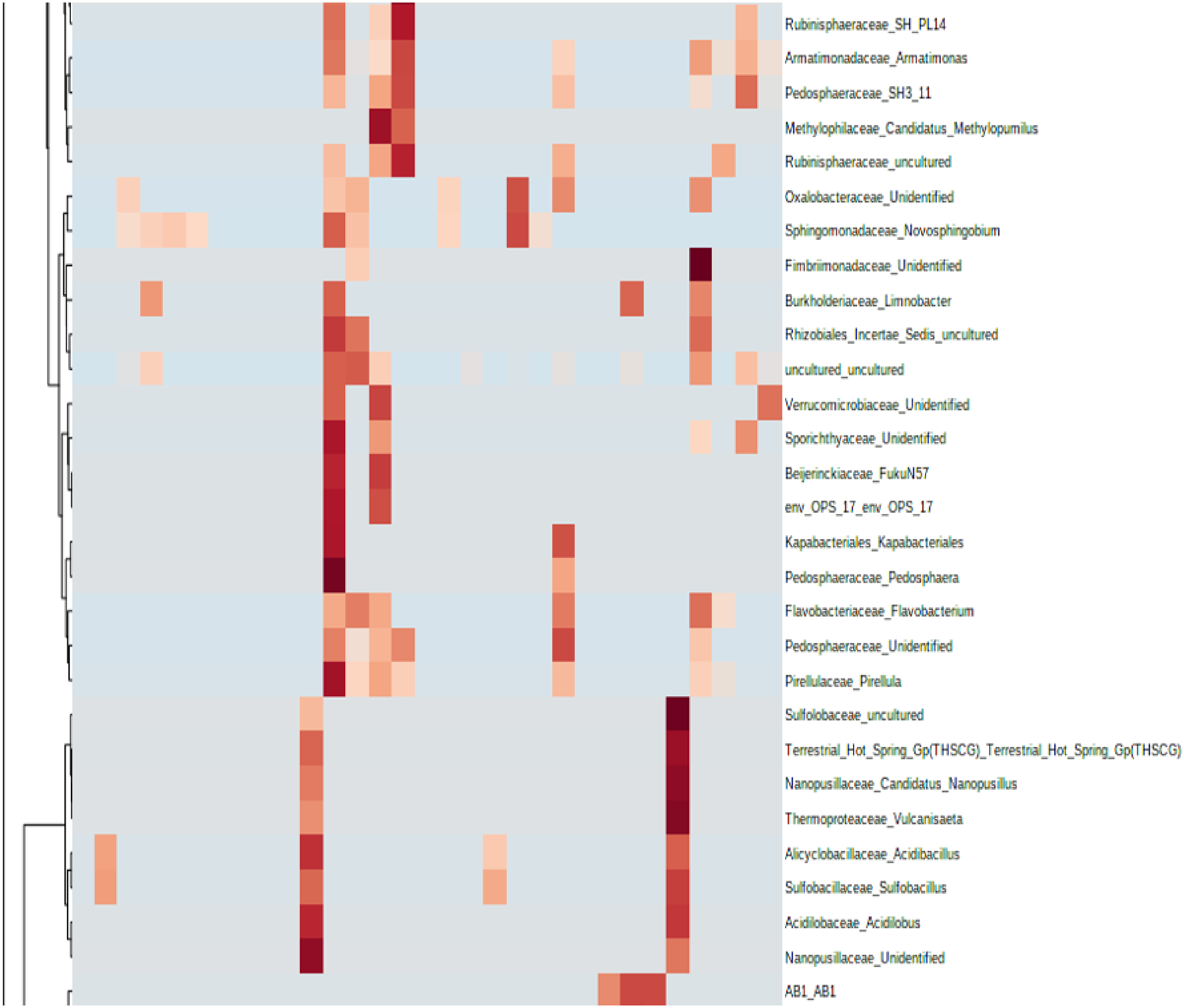
Rubinisphaeraceae_SH_PL14
Armatimonadaceae_Armatimonas
Pedosphaeraceae_SH3_11
Methylophilaceae_Candidatus_Methylopumilus
Rubinisphaeraceae_uncultured
Oxalobacteraceae_Unidentified
Sphingomonadaceae_Novosphingobium
Fimbriimonadaceae_Unidentified
Burkholderiaceae_Limnobacter
Rhizobiales_Incertae_Sedis_uncultured
uncultured_uncultured
Verrucomicrobiaceae_Unidentified
Sporichthyaceae_Unidentified
Beijerinckiaceae_FukuN57
env_OPS_17_env_OPS_17
Kapabacteriales_Kapabacteriales
Pedosphaeraceae_Pedosphaera
Flavobacteriaceae_Flavobacterium
Pedosphaeraceae_Unidentified
Pirellulaceae_Pirellula
Sulfolobaceae_uncultured
Terrestrial_Hot_Spring_Gp(THSCG)_Terrestrial_Hot_Spring_Gp(THSCG)
Nanopusillaceae_Candidatus_Nanopusillus
Thermoproteaceae_Vulcanisaeta
Alicyclobacillaceae_Acidibacillus
Sulfobacillaceae_Sulfobacillus
Acidilobaceae_Acidilobus
Nanopusillaceae_Unidentified
AB1_AB1

2 (B2)

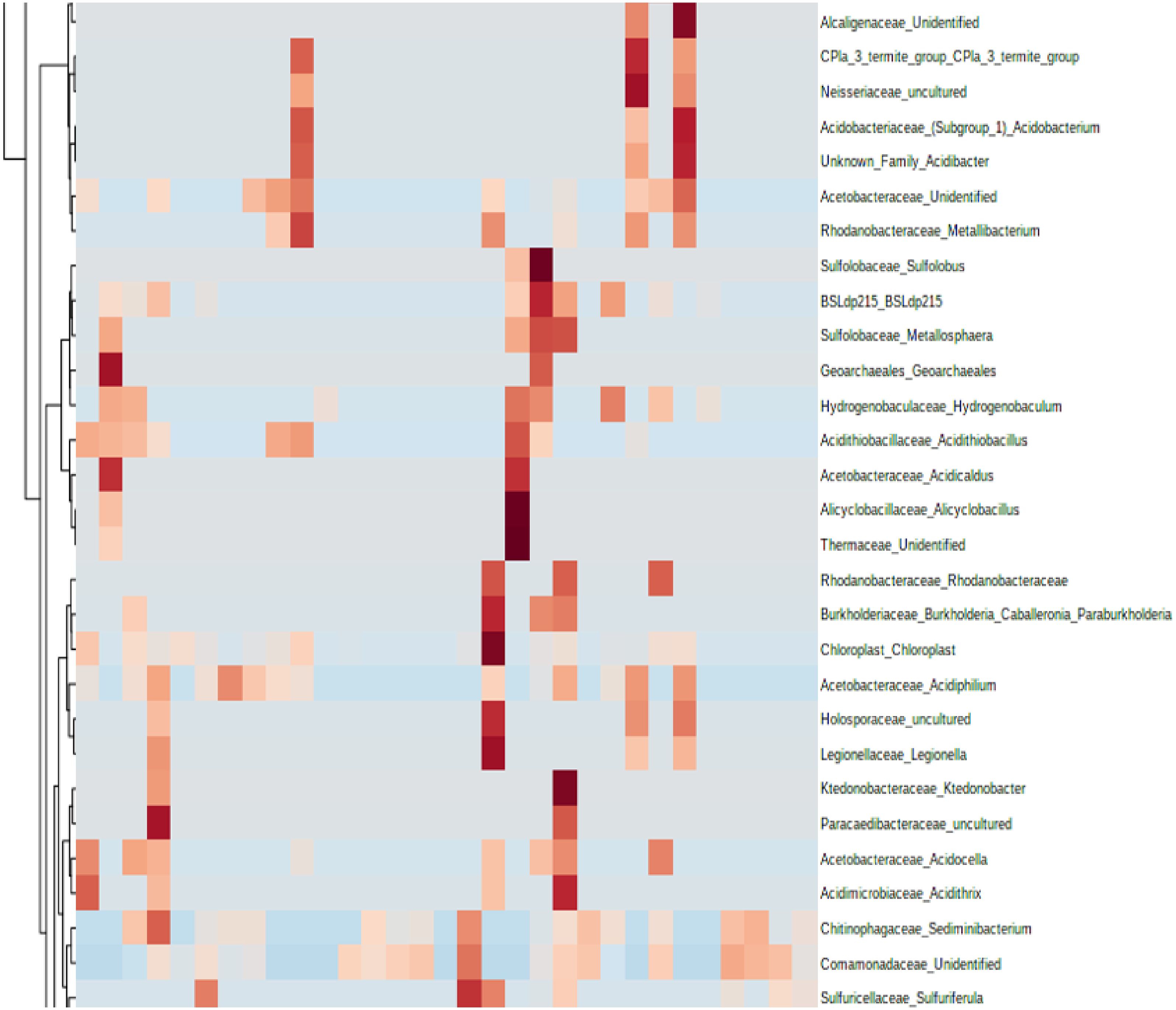
Alcaligenaceae_Unidentified
CPla_3_termite_group_CPla_3_termite_group
Neisseriaceae_uncultured
Acidobacteriaceae_(Subgroup_1)_Acidobacterium
Unknown_Family_Acidibacter
Acetobacteraceae_Unidentified
Rhodanobacteraceae_Metallibacterium
Sulfolobaceae_Sulfolobus
BSLdp215_BSLdp215
Sulfolobaceae_Metallosphaera
Geoarchaeales_Geoarchaeales
Hydrogenobaculaceae_Hydrogenobaculum
Acidithiobacillaceae_Acidithiobacillus
Acetobacteraceae_Acidicaldus
Alicyclobacillaceae_Alicyclobacillus
Thermaceae_Unidentified
Rhodanobacteraceae_Rhodanobacteraceae
Burkholderiaceae_Burkholderia_Caballeronia_Paraburkholderia
Chloroplast_Chloroplast
Acetobacteraceae_Acidiphilium
Holosporaceae_uncultured
Legionellaceae_Legionella
Ktedonobacteraceae_Ktedonobacter
Paracaedibacteraceae_uncultured
Acetobacteraceae_Acidocella
Acidimicrobiaceae_Acidithrix
Chitinophagaceae_Sediminibacterium
Comamonadaceae_Unidentified
Sulfuricellaceae_Sulfuriferula

2(B3)

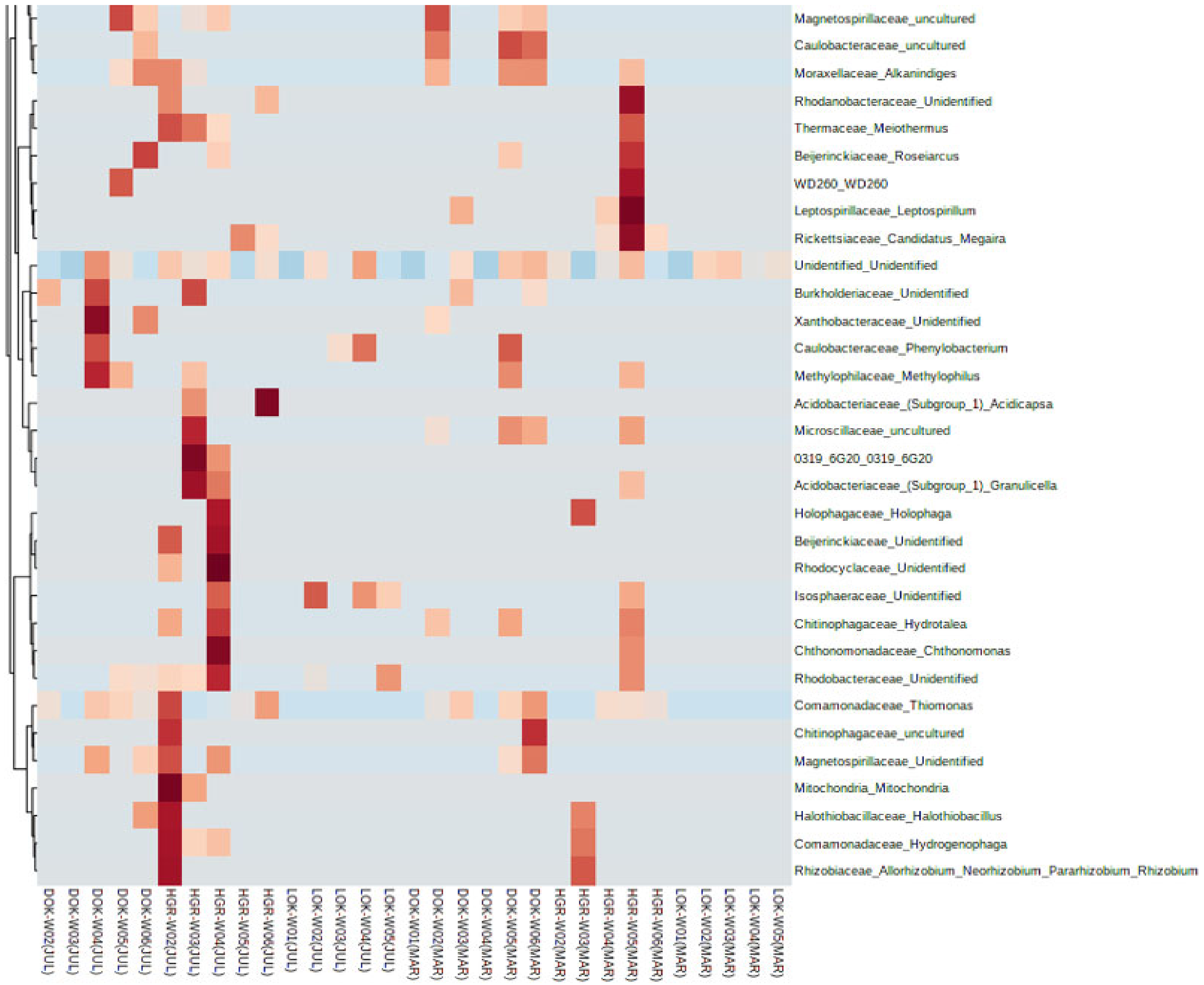

2(B4)

Figure S2(A), 2(B1), 2(B2), 2(B3), 2(B4) Heat map illustrating the abundance and clustering pattern of genera among the three watersheds at two sampling months

Table S1. Geographical location and details of the sampling sites of the three major watersheds of the TVG basin

S.No	Sample number	Sampling location	Latitude	Longitude	First sampling		Second sampling	
					Sampling date	T (°C)	Sampling date	T (°C)
1	PHC-W01*	Dayoukeng	25.172913	121.580031	26.03.2019	50	-	-
2	PHC -W02*	Shanghuangxi parking lot	25.177000	121.583694	26.03.2019	24.2	-	-
3	PHC -W03	Sulphur creek bridge	25.179478	121.582268	26.03.2019	23.2	4.07.2019	25.5
4	PHC -W04	Eightyano creek	25.179478	121.582268	26.03.2019	78	4.07.2019	58.5
5	PHC -W05	Intersection of Bayanxi and Suanxi	25.196388	121.589366	26.03.2019	23.1	4.07.2019	26.2
6	PHC -W06	Sulfur creek middle reach	25.208014	121.593667	26.03.2019	19.8	4.07.2019	25
7	HGC-W01	Xingyi waterfall	25.146530	121.525030	27.03.2019	20	4.07.2019	25.5
8	HGC-W02	Sulphur valley	25.143722	121.521361	27.03.2019	23	4.07.2019	26.7
9	HGC-W03	Quanyuan park	25.141485	121.508386	27.03.2019	24	4.07.2019	27.5
10	HGC-W04	Fuxing park	25.140099	121.503453	27.03.2019	25	4.07.2019	26.5
11	HGC-W05	Wego primary school	25.136361	121.501005	27.03.2019	29	4.07.2019	30
12	HGC-W06	Qiyan pumping station	25.124208	121.503066	27.03.2019	29	4.07.2019	29
13	NHC-W01	Xiaoyoukeng Yongquan	25.175835	121.547494	28.03.2019	75.5	3.07.2019	79.4
14	NHC -W02	Gaojia Banyuechi	25.170080	121.539873	28.03.2019	19	3.07.2019	24.5
15	NHC -W03	Hushan Town	25.160545	121.535683	28.03.2019	22	3.07.2019	24
16	NHC -W04	Dragon Phoenix valley	25.143689	121.528677	28.03.2019	26.5	3.07.2019	27
17	NHC -W05	Imperial pond	25.140989	121.530248	28.03.2019	28.5	3.07.2019	28.5

*Sampled once

Table S2. Physicochemical properties of the hot springs and watersheds located in the TVG basin

Sample Sites	T (°C)	pH	EC	TDS	SO_4^{2-}	Fe	Na	Mg	K	Ca	Zn	Cl^-	Al	As	Pb	Co	Ni	Cd	Reference
**Hot springs**																			
TRK	55.8	1.5	19,540	17,080	3658	28.20	451	61.20	212	213	3.44	2684	404	2.900	1.260	ND	ND	ND	[1]
LHK	50.8	2.8	ND	592	341	2.99	26	19.10	4.82	55.4	0.02	67.0	14.7	0.007	0.006	ND	ND	ND	[2]
LFK	80.3	3.2	295	600	448	24.30	16.70	14.40	5.90	51.6	0.06	24.7	69.6	BD	0.019	ND	ND	ND	[3]
BY	81.2	2.7	2043	772	340	0.56	17.50	9.90	3.50	28.2	14.57	38.1	4.3	ND	ND	ND	ND	ND	[3]
**Watersheds**																			
PHC-US	24.9	4.0	314.1	ND	88.4	0.56	11.49	2.58	2.62	17.6	14.57*	27.5	4.3	0.300*	0.270*	1.07*	0.740*	ND	[4]
PHC-DS	25.1	4.2	255.2	ND	74.8	0.34	10.82	2.39	2.42	16.6	14.84*	25.3	3.9	0.270*	0.310*	1.21*	0.880*	ND	[4]
HGC-US	26.1	6.5	ND	127.7	34.4	0.01	9.73	2.05	2.01	12.2	1.47*	14.8	1.0	1.990*	ND	0.72*	0.710*	ND	[5]
HGC-MS	29.6	3.3	ND	453.1	140.2	0.81	31.36	4.83	11.51	33.1	0.11	85.8	2.8	0.030	30.220*	1.56*	0.005	0.66*	[5]
HGC-DS	30.2	2.5	ND	1354	254.5	6.12	62.25	5.49	26.60	38.6	0.24	214.1	11.9	0.260	85.750*	3.91*	0.020	1.48*	[5]
NHC-US	25.4	6.2	ND	180.2	65.5	5.02*	16.90	5.90	2.83	45.3	1.54*	21.4	169.7*	1.170*	0.590*	0.70*	0.750*	0.11*	[5]
NHC-MS	26.3	6.8	ND	245.4	92.2	60.90*	20.39	6.41	5.58	38.7	1.43*	28.7	212.3*	3.560*	BD	0.90*	1.040*	BD	[5]
NHC-DS	26.8	4.4	ND	296.5	130.5	797*	21.27	5.43	7.09	35.0	22.04*	44.3	2501*	0.840*	0.590*	1.22*	1.740*	0.11*	[5]

Note: EC in μS/cm); elemental concentration in ppm; * the values are expressed in ppb; TRK- Ti-re-ku; LHK-Liou-huang-ku; LFK- Long-feng-ku; BY- Bayan; PHC-Peihuang creek; HGC- Huanggang creek; NHC- Nanhuang creek; US- Upstream; MS- Midstream; DS- Downstream; BD- Below detection limit; ND- No details.

References

1. Hsu, H.-H.; Yeh, H.-F. Factors controlling of thermal water hydrogeochemical characteristics in Tatun Volcano Group, Taiwan. *Water* **2020**, *12*, 2473.
2. Chao, H.-C.; Pi, J.-L.; You, C.-F.; Shieh, Y.-T.; Lu, H.-Y.; Huang, K.-F.; Liu, H.-C.; Chung, C.-H. Hydrogeology constrained by multi-isotopes and volatiles geochemistry of hot springs in Tatun Volcanic Group, Taiwan. *Journal of Hydrology* **2021**, *600*, 126515.
3. Liu, C.-M.; Song, S.-R.; Chen, Y.-L.; Tsao, S. Characteristics and origins of hot springs in the Tatun Volcano Group in Northern Taiwan. *Terrestrial, Atmospheric & Oceanic Sciences* **2011**, *22,* 475-489.
4. Kao, Y.-H.; Wang, S.-W.; Maji, S.K.; Liu, C.-W.; Wang, P.-L.; Chang, F.-J.; Liao, C.-M. Hydrochemical, mineralogical and isotopic investigation of arsenic distribution and mobilization in the Guandu wetland of Taiwan. *Journal of Hydrology* **2013**, *498*, 274-286.
5. Hsiao, Y.H. Hydrochemical Study of Huanggang Creek and South Huangg Creek Using Rare Earth Elements as a Natural Tracer (in Chinese). Master's Thesis, National Chung Cheng University, Minxiong, Chiayi, Taiwan, 2015. (in Chinese).
